# Clinical Outcomes of a Multi-Ingredient *Coriolus versicolor*-Based Vaginal Gel in Greek Women with Low-Grade Cervical Lesions: The PAPILOBS-GR Real-Life Prospective Study

**DOI:** 10.3390/cancers18172762

**Published:** 2026-08-25

**Authors:** Georgios Michail, Alexandros Daponte, George Valasoulis, Konstantinos Dinas, Evripidis Bilirakis, Kalliopi Pappa, Konstantinos Chronopoulos, Christodoulos Akrivis, Zoi Anastasiadi, Marinos Nikolaou, Antonios Garas, Thomas Tsiantis, Antonios Athanasiou, Maria Bertoli, Alexandros Ginis, Evangelos Paraskevaidis

**Affiliations:** 1 Department of Obstetrics and Gynaecology, Faculty of Medicine, School of Health Sciences, University of Patras, General University Hospital of Patras, 26504 Rion, Greece; 2 Department of Obstetrics and Gynaecology, Faculty of Medicine, School of Health Sciences, University of Thessaly, University Hospital of Larissa, 41110 Larissa, Greece; 3 2nd Department of Obstetrics and Gynaecology, Faculty of Medicine, School of Health Sciences, Aristotle University of Thessaloniki, Hippokration General Hospital, 54642 Thessaloniki, Greece; 4 Department of Obstetrics and Gynaecology, IASO Hospital, 15123 Athens, Greece; 5 1st Department of Obstetrics and Gynaecology, Faculty of Medicine, School of Health Sciences, National and Kapodistrian University of Athens, Alexandra University Hospital, 11528 Athens, Greece; 6 Department of Obstetrics and Gynaecology, General Hospital of Argolida-Argos Nursing Unit, 21231 Argos, Greece; 7 Department of Obstetrics and Gynaecology, General Hospital of Ioannina G. Hatzikosta, 45445 Ioannina, Greece; 8 Department of Obstetrics and Gynaecology, General Hospital of Agios Nikolaos, 72100 Agios Nikolaos, Greece; 9 Department of Obstetrics and Gynaecology, Faculty of Medicine, School of Health Sciences, University of Ioannina, University Hospital of Ioannina, 45500 Ioannina, Greece; 10 Medical Department, ELPEN Pharmaceutical Co., Inc., 19009 Attica, Greece

**Keywords:** low-grade cervical lesions, *Coriolus versicolor*, human papillomavirus, real-world evidence, vaginal gel, HPV clearance, cervical lesion regression

## Abstract

**Background**: Persistent human papillomavirus (HPV) can cause low- or high-grade cervical lesions, which may progress to cervical cancer. This prospective, multicenter, real-world study assessed the effectiveness, tolerability and safety of a *Coriolus versicolor*-based vaginal gel for the regression of HPV-related low-grade cervical lesions in routine Greek practice. **Methods**: PAPILOBS-GR is a multicenter, open-label, non-interventional, prospective observational, non-comparative study conducted across 45 sites in Greece. Women aged ≥18 years with ASCUS/LSIL cytology and concordant colposcopy were enrolled. Participants received *Coriolus versicolor*-based vaginal gel for six months. A second 6-month cycle was offered if needed. The primary endpoint was lesion regression. Secondary endpoints included HPV clearance, patient satisfaction, biopsy evolution, tolerability, and safety. **Results**: Of 524 enrolled patients (mean age: 34.4 ± 10.0 years; 40.5% HR-HPV), 494 (94.3%) completed the study. Overall, 75.9% achieved lesion regression (72.1% at 6 months and 34.0% at 12 months). HPV clearance occurred in 72.0% overall (68.1% at 6 months and 50.0% at 12 months). Exploratory comparisons showed generally consistent effectiveness across subgroups, including women with HR-HPV, those aged >40 years, and vaccinated participants. No significant differences in clinical endpoints were observed in association with age or vaccination status. Among 22 patients with baseline and 12-month biopsies, 59.1% showed histological improvement. Satisfaction scores exceeded 8/10 at 6- and 12-month follow-ups. Only two non-serious possibly product-related adverse events were reported. **Conclusions**: *Coriolus versicolor*-based vaginal gel was associated with high rates of lesion regression and excellent tolerability, reinforcing previous clinical findings and supporting its potential role as an adjunctive approach during watchful waiting for HPV-positive low-grade cervical lesions.

## Simple Summary

Low-grade cervical lesions are typically managed through surveillance rather than treatment. However, persistent HPV infection and cervical abnormalities remain a clinical concern for some women, and monitoring alone can be associated with considerable patient anxiety. This observational study expands the clinical evidence for *Coriolus versicolor*-based vaginal gel, a non-hormonal vaginal gel designed to promote tissue regeneration and microbiota balance. Data from this large multicenter cohort in Greece confirmed that a substantial proportion of treated patients achieved lesion regression and HPV clearance. Notably, these clinical benefits were consistent regardless of vaccination status, age, or viral risk level. Despite the limitations of the study, these findings suggest that this vaginal gel may represent a promising adjunctive approach for women with low-grade cervical lesions, while supporting its favorable safety profile. However, further randomized controlled studies are required to confirm its efficacy and define its potential role in clinical practice.

## 1. Introduction

Cervical cancer remains a significant global health burden, ranking as the fifth most common cancer among women worldwide, with approximately 664,000 new cases and 280,000 deaths reported in 2024 [1]. In Greece, it ranks as the 10th most frequent cancer among women overall and the 3rd among women aged 15–44 years [2]. Approximately 282 deaths from cervical cancer are recorded annually in Greece, and in 2020 it was estimated to be the 12th leading cause of cancer-related death among Greek women [2].

Human papillomavirus (HPV) is the etiologic driver of nearly all cervical cancers [3]. Of more than 200 identified HPV genotypes, approximately 14 are classified as high-risk (HR) types [4]. Among these, HR-HPV 16 and 18 account for over 70% of invasive cervical cancer cases globally [3,4]. Recent epidemiological data from a cohort of 3500 Greek women screened between 2021 and 2023 demonstrated an HR-HPV prevalence of 8.8%, with HPV16 being the predominant genotype. Notably, the highest HR-HPV infection rate was observed among women aged 31–35 years (25.5%), highlighting a substantial disease burden in the Greek population [4].

Many HPV infections, and the related low-grade cervical lesions, regress spontaneously, reflecting effective host immune control [5]. HPV activates both local innate and adaptive immunity [5], leading to cytokine-mediated pathways that contribute to viral clearance and lesion regression. However, the precise immunological mechanisms that determine clearance versus persistence remain incompletely understood [5]. A subset of infections becomes persistent, particularly those involving HR-HPV genotypes. Persistence is more frequently observed in older age groups, smokers, non-vaccinated individuals, and those with vaginal dysbiosis. Additional factors associated with persistent infection include coinfection with multiple HPV types, recurrent reinfection, immunosuppression, and prolonged use of oral contraceptives [6,7,8,9,10,11,12]. Persistent HR-HPV infection is the principal driver of cervical carcinogenesis and is strongly associated with progression to high-grade precancerous lesions and, occasionally, to invasive cancer [13,14]. The 5-year risk of progression to cervical intraepithelial neoplasia 3 (CIN3) has been estimated at 20.5% for HR-HPV variants compared with 1.7% for infections caused by low-risk genotypes [15]. Although CIN1 is generally considered as a transient lesion with a high likelihood of spontaneous regression, clinical outcomes vary considerably across patient populations. Infection outcomes are substantially influenced by several host- and virus-related factors, including age, HPV genotype, immune status, and smoking habits. This heterogeneity underscores the importance of accurate risk stratification strategies in HPV management. Among these determinants, age appears to be particularly relevant, as regression rates decline markedly with advancing age, decreasing from 44.7% in younger women to 24.9% in women older than 40 [16].

The natural history of cervical carcinogenesis follows a well-established pathway: persistent HPV infection may lead to low-grade squamous intraepithelial lesions (LSILs) or CIN1, which may progress to high-grade squamous intraepithelial lesions (HSILs) or CIN2-3, and ultimately to invasive carcinoma if left untreated [17]. Current clinical management differs based on lesion grade. For LSIL/CIN1, the standard approach is conservative management with regular cytological, molecular and colposcopic surveillance [18]. However, prolonged follow-up, which may extend over months or years, can impose a significant psychological burden for patients, including anxiety, uncertainty, and reduced quality of life [19,20,21]. In contrast, excisional treatments are recommended for HSIL/CIN2+, most commonly through the loop electrosurgical excision procedure (LEEP) or cold knife conization [18]. While effective, these procedures may not guarantee either complete lesion eradication or HPV clearance. Furthermore, excisional treatments are associated with adverse obstetric outcomes, including an increased risk of preterm birth, low birth weight, and perinatal mortality in subsequent pregnancies [22].

The psychological impact of an HPV-positive diagnosis should not be underestimated. Many women experience negative emotional responses, including anxiety, fear of cancer progression, concerns about sexual transmission, and feelings of stigmatization [21]. This psychological distress can persist long-term and may require additional psychological support, particularly among women undergoing conservative management and prolonged surveillance [20]. The lack of active treatment options during the watchful waiting period may further contribute to patient anxiety and may paradoxically impair immune function, potentially affecting viral clearance [23].

Despite the substantial clinical and psychological burden associated with persistent HPV infection, no pharmacological treatments are currently established to specifically enhance HPV clearance or promote lesion regression. As a result, conservative management remains the standard management strategy for LSIL/CIN1. Nevertheless, adjunctive therapies that accelerate HPV clearance and lesion regression could reduce the need for prolonged surveillance, mitigate patient anxiety, and potentially decrease the risk of progression in selected individuals. Accordingly, increasing attention has been directed toward non-invasive therapeutic options that support host immune function, facilitate cervical epithelial repair, and improve clinical outcomes while avoiding the potential complications associated with surgical interventions [22].

In this context, *Coriolus versicolor*-based vaginal gel (Papilocare^®^, Procare Health, Valencia, Spain) has been evaluated in women with HPV-related low-grade cervical lesions. This vaginal gel combines multiple natural ingredients with complementary biological activities, including components that support tissue moisturization and regeneration (hyaluronic acid, *Centella asiatica*, *Aloe vera*), microbiota-balancing compounds (α-glucan oligosaccharide), and ingredients that have shown beneficial effects on HPV-dependent cervical lesions and viral clearance (*Coriolus versicolor*, *Azadirachta indica*, β-glucan) [24,25,26,27,28]. Clinical evidence further suggests that *Coriolus versicolor*-based vaginal gel may improve cervical re-epithelialization and favorably modulate the vaginal microbiota composition in HPV-positive women [29,30].

Previous randomized controlled trial and observational clinical studies have demonstrated the efficacy, effectiveness and safety of *Coriolus versicolor*-based vaginal gel for cervical lesion repair, cytological and colposcopic normalization, HPV clearance, and overall tolerability across diverse clinical settings [19,31,32,33]. The present study, PAPILOBS-GR, was designed to evaluate the effectiveness, safety, and tolerability of this vaginal gel in a large cohort of Greek women with HPV-related low-grade cervical lesions managed in routine clinical practice. By providing evidence from a distinct geographic and healthcare context, the study aims to further characterize the clinical performance of the treatment under real-world conditions. Additionally, comprehensive descriptive subgroup analyses were conducted to investigate outcomes in clinically relevant populations, including women over 40 years, HPV-vaccinated women, and those harboring HR-HPV infections.

## 2. Materials and Methods

### 2.1. Study Design and Setting

This was a multicenter, open, non-interventional, prospective observational, non-comparative clinical study conducted between September 2021 and April 2023 across Greece (PAPILOBS-GR study, National Clinical Trial Number #NCT06399341; ClinicalTrials.gov; accessed on 25 January 2026).

Patients were recruited from 45 public and private gynecology sites across Greece, including 9 Departments of the public sector (5 University hospital-based, 4 Regional hospital-based, 1 private Maternity hospital-based Colposcopy Department and 35 private gynecology settings, all with established expertise in the follow-up of patients with HPV-related cervical lesions. The complete list of participating sites is provided in Supplementary Table S1. Inclusion criteria included: women aged ≥18 years, vaccinated or not against HPV; a cervical cytology result of atypical squamous cells of undetermined significance (ASCUS) or low-grade squamous intraepithelial lesion (LSIL) performed up to three months prior to inclusion, together with colposcopy findings concordant with cytology results; and an (HR)-HPV molecular test, optionally obtained at most three months prior to study inclusion (the HPV test is typically performed on clinical demand based on physician judgment in Greece; therefore this test was not available for all enrolled women). Exclusion criteria included: any gynecological or non-gynecological condition contraindicating *Coriolus versicolor*-based vaginal gel use [34]; women of childbearing age not using effective contraception; pregnancy, suspected pregnancy, or planning to become pregnant within 6 months; breastfeeding; initiation of vaginal contraceptives (e.g., vaginal ring) or other vaginal hormone treatments during the study; participation in another clinical trial currently or within four weeks prior to inclusion; any planned surgery that would preclude compliance with treatment; and known allergies to any ingredient of *Coriolus versicolor*-based vaginal gel. In addition, all patients were required to read the patient information sheet, sign the informed consent form, and demonstrate willingness to comply with treatment and study procedures to be included in the study.

Eligible patients were enrolled after informed consent. At baseline (Visit 1), assessments included demographics, medical and gynecological history, cervical cytology (Pap test), colposcopy, HPV testing (PCR or validated assays for high-risk strains), and biopsy when clinically indicated per routine practice. Patients received instructions for *Coriolus versicolor*-based vaginal gel application (intravaginal, preferably at bedtime) for appropriate use of the product. The regimen was one vaginal application daily for 21 consecutive days in month 1, then one application on alternate days for the next five months (total initial treatment: 6 months). At 6 months (Visit 2), all patients underwent evaluation with cervical cytology, colposcopy, HPV testing, biopsy if indicated, treatment satisfaction on a 0–10 Likert scale (0 = not at all satisfied, 10 = very satisfied), adherence assessed by direct questioning, and adverse event monitoring. Patients with normal cytology/colposcopy and HPV clearance completed the study at Visit 2; those with persistent abnormalities and/or HPV positivity were offered an additional 6-month treatment with the same regimen, extending total treatment to 12 months. Patients continuing beyond 6 months received a final evaluation with the same assessments at Visit 3.

The study was approved by the corresponding ethics committees (Supplementary Table S2) and conducted in accordance with the Declaration of Helsinki (and subsequent amendments) and Good Clinical and Pharmacoepidemiology Practice guidelines. All participants provided written informed consent before enrollment.

### 2.2. Study Endpoints

The primary endpoint was the percentage of patients with documented regression of HPV-dependent cervical lesions, defined as normalization of cervical cytology (from ASCUS or LSIL at baseline to normal) with concordant normal colposcopy findings at 6 months and/or 12 months, where concordance was classified as normal, non-specific, or abnormal finding grade 1 (minor change) according to the 2011 International Federation of Cervical Pathology and Colposcopy (IFCPC) nomenclature [35]. Secondary endpoints included HPV clearance—measured, where available, by PCR or clinically validated (HR)-HPV detection molecular assays—reported as total clearance (elimination of all baseline genotypes) or partial clearance (elimination of at least one baseline genotype with normalized cytology and colposcopy) at 6 and/or 12 months. HPV testing was conducted according to local clinical practice, and information on the specific assays used was not systematically collected across centers. Other secondary endpoints included patient satisfaction with treatment, assessed using a 0–10 Likert scale (0 = not at all satisfied; 10 = very satisfied) at the same time points; changes in biopsy findings in participants with histology available per routine clinical practice, described as improvement when moving one or more levels along the hierarchy CIN3 → CIN2 → CIN1 → indicates HPV → inflammatory → negative; and safety and tolerability, captured as incidence, nature, severity, and relationship to treatment of adverse events throughout the study.

Primary and secondary endpoints were evaluated after 6 months of treatment, after 12 months when applicable, and overall (6 and/or 12 months). Data was collected in the electronic case report form (eCRF) at baseline via direct questioning and clinical examination, and similarly at the 6- and 12-month visits.

Predefined descriptive subgroup analyses were conducted for the following sub-populations: patients with HR-HPV infection; patients aged ≤40 years versus >40 years; patients >40 years with HR-HPV; vaccinated versus unvaccinated patients; and patients infected with HPV16 and/or HPV18.

To assess the safety of the treatment, a comprehensive record of adverse events throughout the study period was maintained. Descriptive statistics were used to summarize all safety parameters, including adverse events (AEs) and serious adverse events (SAEs), their relationship to the medical device, intensity (mild, moderate, severe), seriousness, and outcomes. Results were presented using tables of absolute and relative frequencies.

### 2.3. Statistical Analysis

Sample size justification: The planned sample size was 800 participants, based on an expected cervical normalization rate of 65%, a precision of ±3.5 percentage points (95% confidence interval), and 10% attrition. A total of 524 participants were enrolled and 491 were evaluable. Given the observed cervical normalization rate of 75.9%, a minimum of 487 evaluable patients would have been required to achieve a precision of ±3.8 percentage points. Therefore, the final sample of 491 evaluable patients achieved precision comparable to that originally planned for the primary endpoint.

Categorical variables were expressed as absolute and relative frequencies. Continuous variables were presented as measures of central tendency (mean and median) and measures of dispersion (standard deviation, minimum, and maximum), including the total number of valid values and 95% confidence intervals.

Distribution of quantitative variables was assessed using both statistical tests (Kolmogorov–Smirnov or Shapiro–Wilk tests) and graphical approaches (histograms, Q-Q plots). For normally distributed data, parametric tests were used (independent-sample *t*-test, paired *t*-test, ANOVA). Non-normally distributed data were compared using non-parametric tests (Mann–Whitney U test, Wilcoxon signed-rank test, Kruskal–Wallis test). For comparison of categorical variables, chi-square test, McNemar test, or Fisher’s exact test were implemented as appropriate.

Statistical significance was set at *p* ≤ 0.05 (two-sided). All statistical analyses were performed using IBM SPSS software version 28.0 (IBM Corp., Armonk, NY, USA) or RStudio version 2023.06.0 (RStudio, PBC, Boston, MA, USA).

## 3. Results

### 3.1. Patient Disposition and Baseline Characteristics

**Figure 1:**
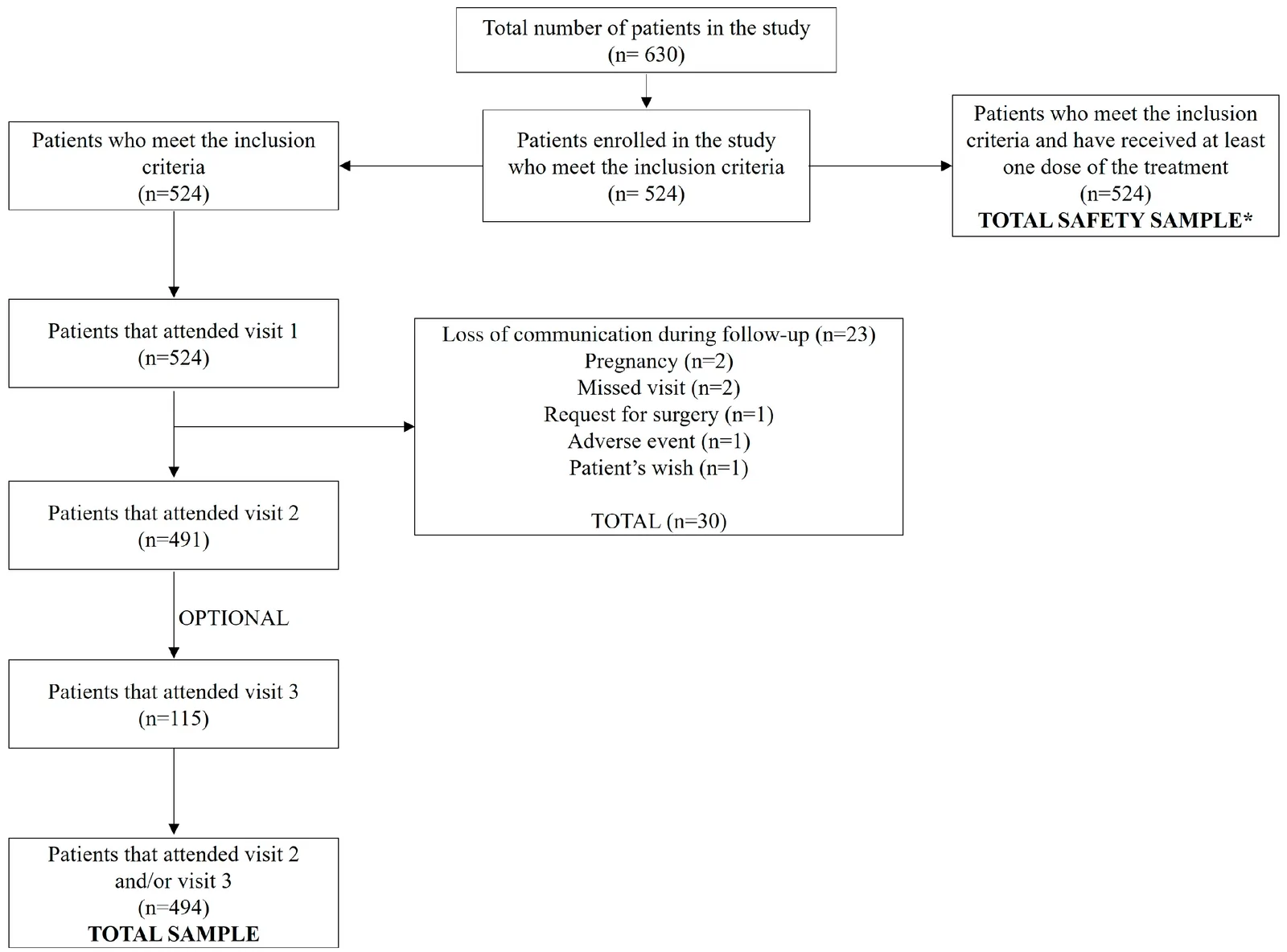
Patient flow chart. * Safety population of the study includes all patients who met the inclusion criteria, who entered the study and who received at least one dose of the treatment under consideration.

A total of 524 patients were enrolled across 45 Greek sites. Of these, 494 patients (94.3%) completed the study in 6 or 12 months and comprised the total analysis sample. Thirty patients (5.7%) discontinued the study for the following reasons: loss of communication during follow-up (n = 23, 4.4%), pregnancy (n = 2, 0.4%), a missed visit due to non-compliance (n = 2, 0.4%), patient request for surgery (n = 1, 0.2%), adverse events (n = 1, 0.2%), and the patient’s wish (n = 1, 0.2%). All 524 enrolled patients who received at least one treatment application were included in the safety analysis. The patient flow is shown in Figure 1.

Baseline demographic and clinical characteristics of the total sample are presented in Table 1. The mean age was 34.4 ± 10.0 years, with 393 patients (75.0%) aged ≤40 years and 131 (25.0%) aged >40 years. Most participants were Caucasian (509, 97.1%). Regarding cytology results, 332 patients (63.4%) had LSIL and 192 (36.6%) had ASCUS at baseline.

HPV testing data were available for 321 patients (61.3%) at baseline. Among the 321 patients with available HPV data, 212 had HR-HPV infection (40.5%), and 57 (26.9%) were infected with HPV16 and/or HPV18.

Regarding HPV vaccination status, 121 patients (23.1%) had been vaccinated against HPV, while 403 (76.9%) were unvaccinated. Concerning smoking habits, 170 patients (32.4%) were current smokers, 76 (14.5%) were ex-smokers, and 278 (53.1%) were non-smokers at baseline.

Baseline biopsy results were available for 137 patients (26.1%). Among these, the most frequent histological result was CIN1 (n = 107, 78.1% of biopsies), followed by findings indicating HPV (n = 18, 13.1% of biopsies), inflammatory changes (n = 8, 5.8% of biopsies), and negative results (n = 4, 2.9% of biopsies). No CIN2 or CIN3 lesions were detected at baseline.

### 3.2. Primary Endpoint: Regression of HPV-Induced Cervical Lesions

Overall, the vaginal gel managed to achieve cervical lesion regression in 75.9% of participants (95% CI: 72.1–79.7%). After 6 months (Visit 2), 72.1% had cervical lesion regression (95% CI: 68.1–76.1%), and among those with persistent lesions at Visit 2, a further 34.0% achieved lesion regression by 12 months (Visit 3; 95% CI: 21.2–46.7%).

Subgroup analyses demonstrated consistent efficacy across all predefined populations. In patients with HR-HPV, cervical lesion regression was observed in 69.4% overall (95% CI: 62.9–75.9%); at Visit 2 and Visit 3, lesion regression rates were 68.9% (95% CI: 62.4–75.4%) and 9.1% (95% CI: 0.0–26.1%), respectively.

When stratified by age, overall lesion regression among women older than 40 years reached 78.4% (95% CI: 71.2–85.6%), with 72.0% at Visit 2 (95% CI: 64.1–79.9%) and 44.4% at Visit 3 (95% CI: 21.5–67.4%). In those patients aged ≤40 years, lesion regression was 75.0% overall (95% CI: 70.5–79.5%), with 72.2% at Visit 2 (95% CI: 67.5–76.8%) and an additional 28.6% at Visit 3 (95% CI: 13.6–43.5%). Chi-square statistical comparisons between ≤40 and >40 years showed no significant differences at Visit 2 (*p* = 1.000), Visit 3 (*p* = 0.359), or overall, across the study (*p* = 0.469).

In the subgroup of patients >40 years with HR-HPV, 62.2% achieved lesion regression overall (95% CI: 46.5–77.8%); lesion regression occurred at Visit 2, with no additional regressions observed at Visit 3.

By vaccination status, lesion regression was achieved overall in 76.6% of vaccinated patients (95% CI: 68.6–84.7%) and 75.7% of unvaccinated patients (95% CI: 71.3–80.0%); at Visit 2 and Visit 3; lesion regression rates were 73.8% (95% CI: 65.5–85.2%) and 33.3% (95% CI: 2.5–64.1%) in vaccinated patients, and 71.7% (95% CI: 67.1–76.2%) and 34.1% (95% CI: 20.1–48.1%) in unvaccinated patients, respectively. Statistical comparisons between vaccinated and unvaccinated showed no significant differences at Visit 2 (*p* = 0.715), Visit 3 (*p* = 0.127), or overall, across the study (*p* = 0.898).

**Figure 2:**
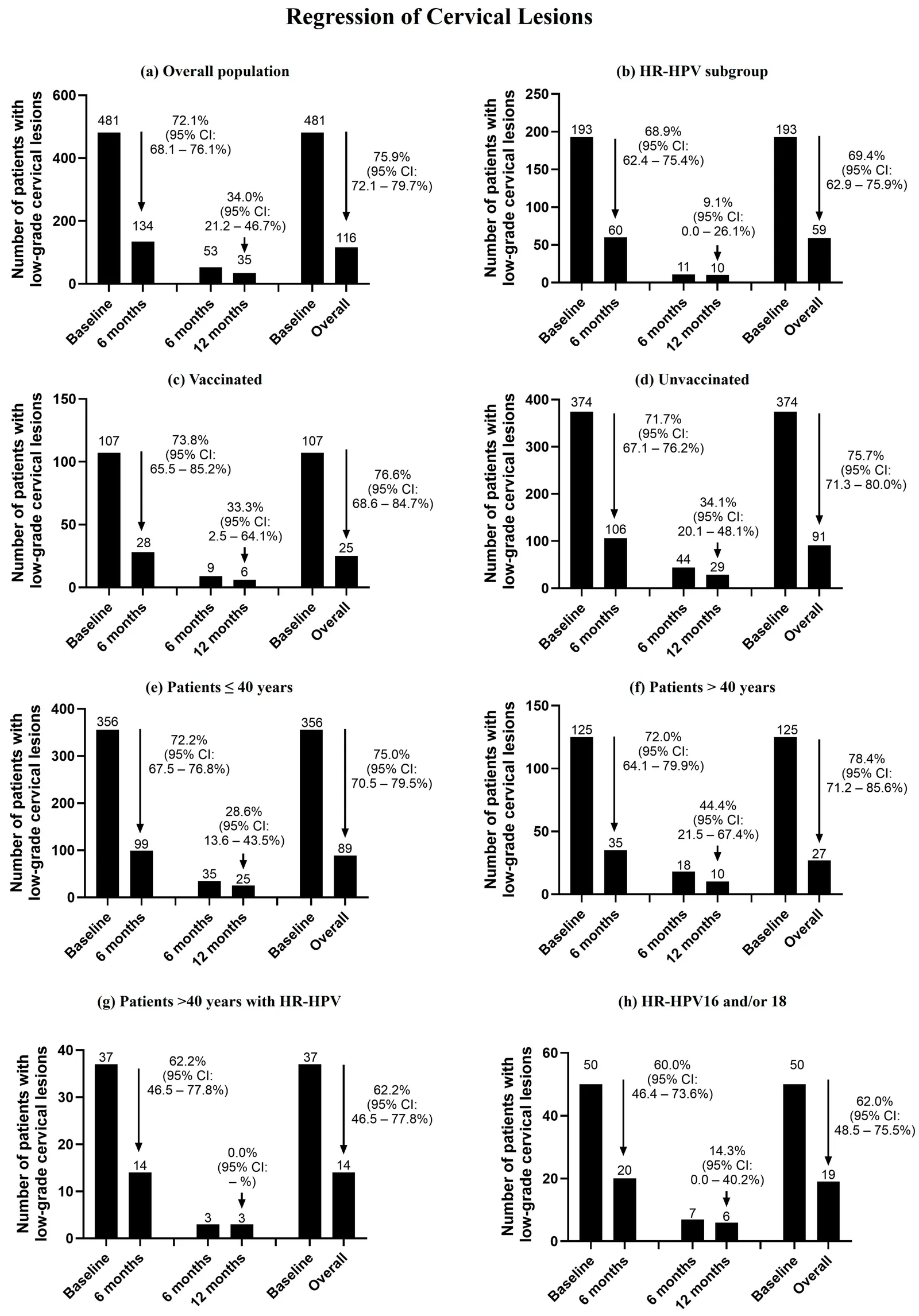
HPV-induced cervical lesion regression rates in overall sample and subgroups. (**a**) Overall population. (**b**) HR-HPV subgroup. (**c**) Vaccinated individuals. (**d**) Unvaccinated individuals. (**e**) Patients aged 40 or younger. (**f**) Patients older than 40. (**g**) Patients older than 40 infected with HR-HPV. (**h**) HPV16 and/or 18 subgroup. Bars represent number of patients with low-grade cervical lesions at baseline, 6 months, 12 months, and overall study. CI, confidence interval; HPV, human papillomavirus; HR, high-risk. Participants with missing data on the primary endpoint at 6 months were excluded from the analysis.

In patients infected with HPV16 and/or HPV18, overall cervical lesion regression was 62.0% (95% CI: 48.5–75.5%); Visit 2 and Visit 3 regression rates were 60.0% (95% CI: 46.4–73.6%) and 14.3% (95% CI: 0.0–40.2%), respectively (Figure 2).

### 3.3. Secondary Endpoint: HPV Clearance

HPV clearance analysis was performed in patients with available HPV testing results at both baseline and follow-ups. Overall, 236 of the 321 patients with baseline HPV data were included in this analysis. Overall, 72.0% achieved clearance by study end (95% CI: 66.3–77.8%). At 6 months (Visit 2), clearance was documented in 68.1% (95% CI: 62.1–74.1%); among those still HPV-positive at Visit 2 who continued therapy, a further 50.0% achieved clearance by 12 months (Visit 3; 95% CI: 30.0–70.0%).

In the HR-HPV subgroup, overall clearance reached 67.3% (95% CI: 60.2–74.3%), with 66.5% at Visit 2 (95% CI: 59.3–73.6%) and an additional 26.7% at Visit 3 (95% CI: 4.3–49.9%).

Age-stratified analyses showed comparable outcomes: among patients ≤40 years, overall clearance was 70.9% (95% CI: 64.3–77.5%), with 68.5% at Visit 2 (95% CI: 61.7–75.4%) and 36.8% at Visit 3 (95% CI: 15.2–58.5%); among those >40 years, overall clearance was 75.9% (95% CI: 64.5–87.3%), with 66.7% at Visit 2 (95% CI: 54.1–79.2%) and 71.4% at Visit 3 (95% CI: 38.0–100.0%). Chi-square tests indicated no statistically significant differences between ≤40 and >40 years at Visit 2 (*p* = 0.868), Visit 3 (*p* = 0.381), or overall (*p* = 0.496).

In patients >40 years with HR-HPV, overall clearance was 67.7% (95% CI: 51.3–84.2%), with clearances occurring by Visit 2 and no additional conversions at Visit 3.

By vaccination status, overall clearance was 65.1% in vaccinated patients (95% CI: 50.9–79.4%) and 73.6% in unvaccinated patients (95% CI: 67.4–79.8%); corresponding Visit 2/Visit 3 rates were 60.5%/40.0% (95% CI: 45.9–75.1% and 0.0–82.9%) in vaccinated and 69.8%/47.6% (95% CI: 63.3–76.4% and 26.3–69.0%) in unvaccinated patients. Chi-square tests indicated no statistically significant differences between vaccinated and unvaccinated at Visit 2 (*p* = 0.277), Visit 3 (*p* ≥ 0.999), or overall (*p* = 0.265).

**Figure 3:**
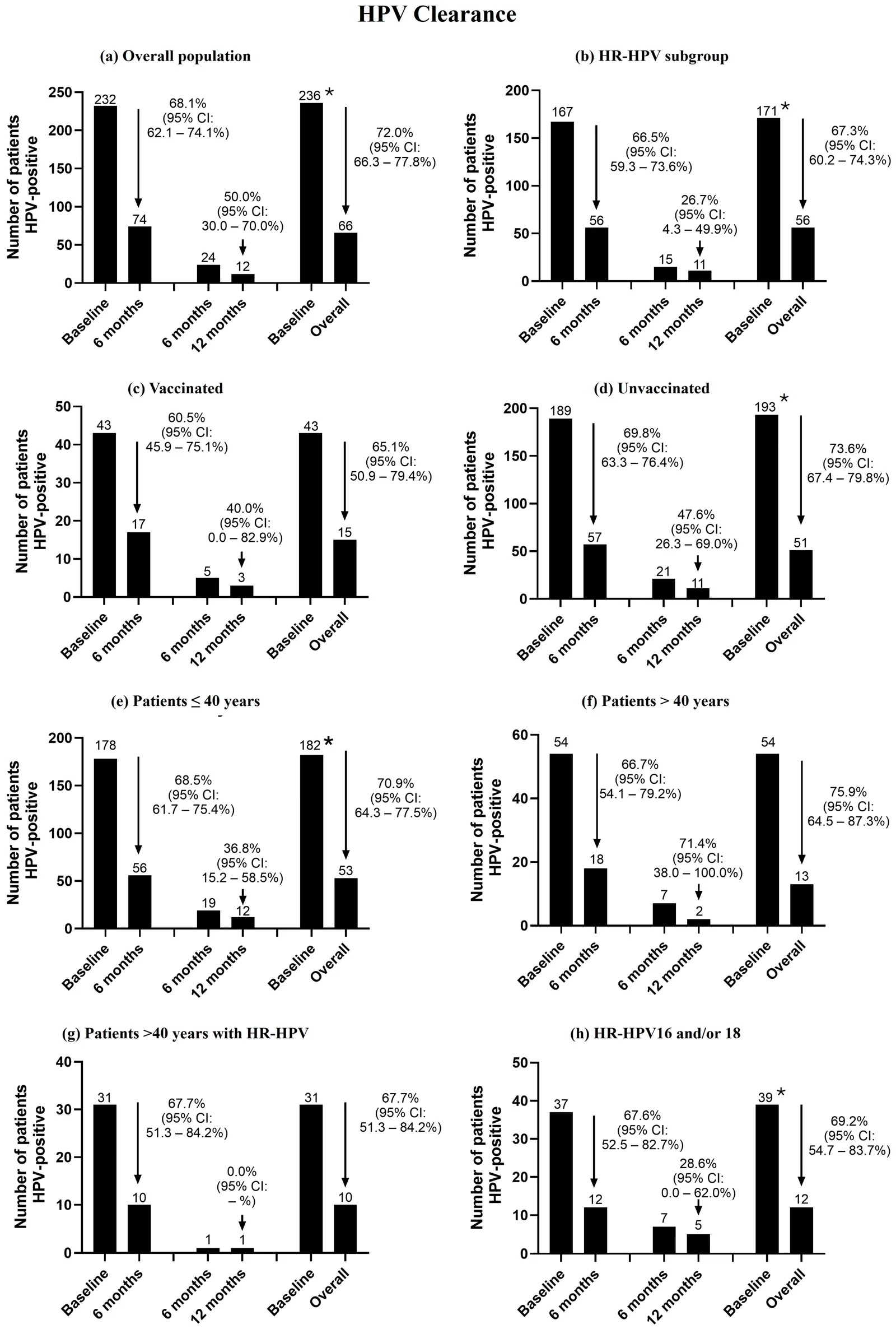
Partial or total HPV clearance rates in overall sample and subgroups. (**a**) Overall population. (**b**) HR-HPV subgroup. (**c**) Vaccinated individuals. (**d**) Unvaccinated individuals. (**e**) Patients aged 40 or younger. (**f**) Patients older than 40. (**g**) Patients older than 40 infected with HR-HPV. (**h**) HPV16 and/or 18 subgroup. Bars represent number of HPV-positive patients at baseline, 6 months, 12 months, and overall study. CI, confidence interval; HPV, human papillomavirus; HR, high-risk. * Patients with test results available at baseline (and 12 months). Some patients missed HPV testing at 6 months but were tested at 12 months.

Among those patients infected with HPV16/18, overall clearance was 69.2% (95% CI: 54.7–83.7%); at Visit 2 and Visit 3, clearance was 67.6% (95% CI: 52.5–82.7%) and 28.6% (95% CI: 0.0–62.0%), respectively (Figure 3).

### 3.4. Evaluation of Changes in Biopsy Findings

Baseline biopsy results were available for 137 patients (26.1% of the total sample). Most showed CIN1 (n = 107, 78.1% of biopsies), followed by findings indicating HPV (n = 18, 13.1%), inflammatory changes (n = 8, 5.8%), and negative histology (n = 4, 2.9%).

Follow-up histological data were limited. Although 127 patients without a negative biopsy result at baseline attended the 6-month visit, only 17 underwent repeat biopsy. At the 12-month visit, 17 patients underwent biopsy, but only 5 underwent biopsy at both the 6- and 12-month assessments. At 6 months, 4/17 (23.5%) improved relative to baseline. Specifically, of two individuals with CIN1 histology and two with “HPV-indicative” histology shifted to “inflammatory”. In addition, 2 (11.8%) with CIN1 at baseline worsened, with one progressing to CIN2 and the other to CIN3. The remaining 11 out of 17 (64.7%) had unchanged histology results (8 persisted as CIN1; two persisted as “HPV-indicative”, and one persisted as negative).

At 12 months, 10/17 (58.8%) improved relative to baseline. Specifically, of seven individuals with CIN1 at baseline, 6 shifted to “HPV-indicative” and one to “inflammatory”. Of 3 with “HPV-indicative” at baseline, one normalized and 2 shifted to “inflammatory”. The remaining 7 out of 17 (41.2%) had unchanged results (6 persisted as CIN1; one persisted as “HPV-indicative”).

Only five patients were biopsied at both 6 and 12 months; three (60.0%) improved between visits (all three regressed from CIN1 at 6 months to “inflammatory” [n = 1] or “HPV-indicative” [n = 2] at 12 months), while two (40.0%) had unchanged results (“CIN1” and “HPV-indicative,” respectively).

Overall, there were 22 patients with biopsy results at 12 months. Of these 22 patients, 13 (59.1%) demonstrated histological improvement.

### 3.5. Patient Satisfaction

Patient-reported satisfaction remained high across assessments on the 0–10 Likert scale. At 6 months (Visit 2; n = 491), the mean score was 8.3 ± 1.7 (median: 9; range: 2–10; IQR: 2). At 12 months (Visit 3; n = 115), the mean was 8.6 ± 1.6 (median: 9; range: 3–10; IQR: 2) (Table 2). Thus, among the patients who had not met the primary endpoint at 6 months and continued treatment to 12 months, satisfaction scores shifted upward (*p* = 0.005).

### 3.6. Smoking Behavior Changes

A subset of patients achieved smoking cessation during the study period. This behavioral change is noteworthy given the established relationship between smoking and immune function, which might affect HPV clearance rates observed in this cohort.

At baseline, more than half of the patients were non-smokers (278/524, 53.1%), while 32.4% (170/524) and 14.5% (76/524) were smokers and ex-smokers, respectively. At Visit 2 (6 months, n = 491), most patients were non-smokers (323, 65.8%), while 117 (23.8%) continued smoking and 51 (10.4%) quit smoking since baseline. Notably, among the 170 patients who were smokers at baseline, the smoking cessation rate was 30.0% (51/170) at 6 months. By Visit 3 (12 months, n = 115), the distribution remained relatively stable, with 74 (64.3%) non-smokers, 32 (27.8%) active smokers, and 9 (7.8%) who had successfully quit smoking.

### 3.7. Safety and Tolerability

Among the 524 patients in the safety population, only 2 patients (0.4%) experienced adverse events, both of which were non-serious and occurred shortly after the baseline visit. One patient reported vaginal burn and tingle sensation (0.2%), and another patient reported vaginal internal itching (0.2%). Both adverse events were classified as related or possibly related to treatment. One (1) patient discontinued the study due to an adverse event and did not attend the subsequent follow-up visits at 6 months (Visit 2) and 12 months (Visit 3). No other adverse events or serious adverse events were reported throughout the entire study period.

## 4. Discussion

This large, multicenter, prospective observational study conducted across 45 Greek sites shows that *Coriolus versicolor*-based vaginal gel was associated with high rates of low-grade cervical lesion regression and HPV clearance under real-world clinical practice conditions. The overall cervical lesion regression rate of 75.9% observed in PAPILOBS-GR substantially exceeds spontaneous regression rates reported in the literature. Historical data indicate that approximately 35–40% of women with CIN1 achieve spontaneous regression within one year, and 40–60% within two years [36,37,38]. Matsumoto et al., in a large prospective study of 570 women with LSIL cytology and a mean age of 36.0 years (comparable to PAPILOBS-GR’s 34.4 years), reported a cumulative two-year regression rate of 62.3% [15]. Notably, the 6-month lesion regression rate observed in PAPILOBS-GR (72.1%) is greater than cumulative two-year spontaneous regression rate reported by Matsumoto et al. However, direct comparisons should be interpreted with caution given the differences in study design, patient populations, and outcome assessment methods.

The treatment benefit becomes even more pronounced when considering age-related factors. During observational management, Bekos et al. demonstrated that spontaneous regression rates decline dramatically with age, decreasing from 44.7% in women under 25 years to only 24.9% in women over 40 years [16]. In PAPILOBS-GR, women aged over 40 achieved a lesion regression rate of 78.4%, more than three times higher than the spontaneous regression rates previously reported for this age group. This finding is particularly noteworthy, as older age is consistently associated with increased HPV persistence and higher risk of progression due to age-related immunosenescence [39].

While cervical lesion regression was the primary endpoint of the PAPILOBS-GR study, HPV clearance represents a particularly relevant endpoint as a more objective and clinically meaningful outcome. HPV testing offers superior sensitivity (>95% vs. 50–70% for cytology), stronger negative predictive value (>99%), and reduced inter-observer variability [40,41]. Most importantly, persistent HR-HPV infection is the single most important risk factor for progression to high-grade cervical lesions and invasive cervical cancer [13,14,42]. The PAPILOBS-GR overall HPV clearance rate of 72.0% exceeds typical spontaneous clearance rates reported in the literature (20–50% at 12 months) [43]. Remarkably, the 67.3% clearance rate in HR-HPV infections and 69.2% in HPV16/18-positive patients are higher than spontaneous clearance rates previously reported for these persistent genotypes (40–63% over 12 months in young women) [44]. HPV genotype distribution varies geographically and may influence both viral persistence and the risk of progression. In Greece, HPV16 is the most prevalent high-risk genotype, followed by HPV31, while HPV51, HPV56, HPV58, HPV66, and HPV45 are also commonly detected [4]. Differences in the prevalence and oncogenic potential of specific HPV genotypes across populations have led to increasing interest in extended HPV genotyping for risk stratification and optimization of clinical management [45]. Although HPV16 and HPV18 status were evaluated in the present study, extended genotyping data were not systematically available, precluding a more comprehensive assessment of genotype-specific treatment outcomes. By promoting clearance of high-risk genotypes, Papilocare^®^ may contribute to reducing viral persistence, a key factor in the development and progression of HPV-dependent lesions. Although the present study was not designed to evaluate progression to high-grade cervical lesions, reducing persistent HR-HPV infection could potentially have favorable implications for long-term cervical outcomes. In turn, this may contribute to reducing the need for excisional treatment, thereby minimizing associated obstetric risks [22]. The achievement of both lesion regression and HPV clearance in approximately 60–65% of patients suggests a comprehensive response addressing both clinical manifestation and underlying viral cause. This dual benefit may have important implications for patient well-being, psychological burden and overall quality of life.

The consistently high lesion regression and HPV clearance rates observed across all subgroups, including those traditionally considered at increased risk of persistence (women aged >40 years, unvaccinated individuals, and those with HR-HPV or HPV16/18 infections), further support a treatment-associated effect across clinically challenging patient populations. The absence of significant age-related differences in both outcomes contrasts with the well-documented age-associated decline in spontaneous lesion regression and clearance rates [16,39], suggesting that the effectiveness of *Coriolus versicolor*-based vaginal gel may be maintained despite age-related factors that contribute to HPV persistence.

The PAPILOBS-GR results closely align with previous clinical studies, providing additional evidence across different settings and populations. The Spanish PAPILOBS study (192 patients) reported 77.1% lesion regression and 71.6% HPV clearance rates, which were remarkably similar to the 75.9% and 72.0% rates observed in PAPILOBS-GR’s despite minor differences in population characteristics, including mean age (38.7 vs. 34.4 years) and vaccination rate (17.6% vs. 23.1%) [33]. The nearly identical HPV clearance rates observed across both studies, despite marked differences in the proportion of patients undergoing HR-HPV testing (93.2% vs. 40.5%), further support the reproducibility of *Coriolus versicolor*-based vaginal gel’s effect.

The PALOMA randomized controlled trial demonstrated an 84.9% lesion regression rate in the treatment group vs. 64.5% in controls (*p* = 0.031) [19]. The slightly higher regression rate observed in PALOMA compared with the 6-month rate of 72.1% in PAPILOBS-GR’s may reflect differences between controlled clinical trial and real-world routine clinical practice. The PALOMA study also showed a trend toward higher HPV clearance in treated patients (59.6% vs. 41.9%, *p* = 0.118), likely not reaching statistical significance. Notably, a sub-analysis of PALOMA focusing on women over 40 years confirmed sustained efficacy of *Coriolus versicolor*-based vaginal gel in this age group [32], consistent with the results obtained in PAPILOBS-GR. Additional evidence comes from a retrospective Italian observational study by Criscuolo et al., which evaluated 183 HR-HPV-positive patients and reported HPV clearance rates of 67.0% in the *Coriolus versicolor*-based vaginal gel group compared to 37.2% in controls (*p* < 0.0001) after 6 months [31]. This study also demonstrated colposcopy improvement in 76.1% of treated women versus 40.8% in controls (*p* = 0.0005). The HPV clearance rate observed in the Italian study (67.0%) is nearly identical to the PAPILOBS-GR 6-month clearance rate of 68.1%, despite different study designs and populations. Further support is provided by a recent observational study conducted in a Turkish population by Haticegul et al. In this study, compared with conservative management, treatment with *Coriolus versicolor*-based vaginal gel was associated with significantly higher rates of cytological normalization (88.4% vs. 60.4%, *p* < 0.001) and HR-HPV clearance (77.0% vs. 25.4%, *p* < 0.001) [46]. This remarkable convergence of findings across studies employing different designs, including randomized controlled trials and prospective and retrospective observational studies, conducted in different countries (Spain, Italy, Greece, Turkey), and involving populations with varying demographic and clinical characteristics, strongly supports the consistency and generalizability of *Coriolus versicolor*-based vaginal gel’s effectiveness. Collectively, these data reinforce the potential clinical role of this adjunctive approach in promoting cervical lesion regression and HPV viral clearance.

In PAPILOBS-GR, 23.1% of patients had been vaccinated against HPV, yet the outcomes of the *Coriolus versicolor*-based vaginal gel showed no significant differences between vaccinated and non-vaccinated patients. This highlights the continued need for effective management strategies in women with established HPV infection. This is particularly relevant given that currently available vaccines are purely prophylactic, do not treat existing infections, and not provide complete coverage against all oncogenic HPV types [47]. The clinical outcomes of the *Coriolus versicolor*-based vaginal gel in non-vaccinated women may be particularly relevant for settings with lower vaccination coverage and where effective non-invasive management options for established HPV infection remain an important clinical need.

Smoking is a well-established cofactor in HPV persistence and cervical cancer development [48]. In the real-world study settings of PAPILOBS-GR, 32.4% of patients were current smokers at baseline, and approximately 30% of these individuals reported smoking cessation by the 6-month visit. While this raises considerations about potential confounding, high overall success rates in lesion and HPV-related outcomes were observed despite relatively high prevalence of smokers in the cohort; suggesting that *Coriolus versicolor*-based vaginal gel can be effective even in the presence of this negative prognostic factor. From a clinical perspective, the observed smoking cessation during the study is also noteworthy. Despite the reasons underlying this change cannot be established from the present study, participation in a structured treatment program may have encouraged positive lifestyle changes, representing an additional benefit beyond clinical outcomes.

The safety profile of *Coriolus versicolor*-based vaginal gel was excellent, with only two mild-to-moderate product-related adverse events in 524 patients (0.4%). Patient satisfaction remained high throughout treatment (mean scores of 8.3/10 and 8.6/10 at 6 and 12 months, respectively), while the high completion rate (93.7%) and minimal discontinuation rate support the acceptability and tolerability of this intervention in routine clinical practice.

In summary, the PAPILOBS-GR study demonstrates that *Coriolus versicolor*-based vaginal gel may represent an effective adjunctive option for the management of low-grade cervical lesions in routine clinical practice. Beneficial outcomes in terms of lesion regression and HPV clearance were observed across all patient subgroups, including populations considered at increased risk of persistence. The favorable safety profile, high patient satisfaction, and good tolerability further support the potential clinical utility of this non-invasive treatment as an adjunctive management option during the watchful waiting period.

Key strengths of PAPILOBS-GR include its large sample size (524 enrolled, 491 completers), multicenter design across diverse clinical settings, real-world observational approach enhancing external validity, high completion rate (93.7%), comprehensive exploratory subgroup analyses, and validation of findings in a different healthcare context. However, several limitations must be acknowledged. The absence of a control group precludes definitive attribution of the observed outcomes solely to treatment, though the magnitude, timing, and consistency of benefit across patient subgroups argue a potential treatment-associated therapeutic effect. Recruitment during the COVID-19 pandemic (September 2021–April 2023) affected enrollment and follow-up procedures. The primary endpoint was based on cytological and colposcopic assessments, both of which are subject to interobserver variability. Histological outcomes were available for only a limited number of participants, and HPV testing and genotyping were not systematically performed in all enrolled women, resulting in incomplete data for the HPV-related secondary endpoint. In addition, the duration of HPV infection before study entry was unavailable, precluding the distinction between transient and persistent infections. Furthermore, as a multicenter observational study conducted under real-world conditions, some variability in diagnostic procedures and HPV assays across participating centers was inevitable. Finally, the relatively short 12-month follow-up prevents assessment of the long-term durability and recurrence rates. Despite these limitations, the consistency of the findings with those reported in previous controlled and observational studies [19,31,33,46], together with the positive clinical outcomes, biological plausibility of the mechanism of action, and excellent safety profile, support the potential clinical value of *Coriolus versicolor*-based vaginal gel in women with HPV-dependent low-grade cervical lesions.

Future research should prioritize randomized controlled trials involving broader populations and diverse healthcare settings to further evaluate the efficacy of *Coriolus versicolor*-based vaginal gel, as well as long-term follow-up studies to assess the durability of response and recurrence patterns. Additional areas of interest include cost-effectiveness analyses, comparative effectiveness research, investigations in women with persistent HPV infection incorporating comprehensive genotyping data, and evaluation of its potential role in the management of CIN2/HSIL or in the prevention of recurrence following excisional treatment. Such studies may help define the overall clinical value of *Coriolus versicolor*-based vaginal gel within the management of HPV-related disease.

## 5. Conclusions

Current management of low-grade cervical lesions relies primarily on conservative surveillance, an approach that often subjects patients to prolonged surveillance (12–24 months or longer) and may be associated with considerable psychological burden. In the present study, *Coriolus versicolor*-based vaginal gel was associated with high rates of cervical lesion regression and HPV clearance within 6–12 months, while demonstrating a favorable safety and tolerability profile. *Coriolus versicolor*-based vaginal gel may represent a valuable adjunctive non-invasive option for women with HPV-related low-grade cervical lesions, including those aged over 40 years and those infected with HR-HPV genotypes.

## Figures and Tables

**Table 1:** Baseline characteristics of the total sample (n = 524).

Characteristic	Value
**Demographics**	
Age, mean years (SD)	34.4 (10.0)
Age ≤ 40 years, n (%)	393 (75.0)
Age > 40 years, n (%)	131 (25.0)
Ethnicity, n (%)	
- Caucasian	509 (97.1)
- Asian	1 (0.2)
- Other	14 (2.7)
**Clinical Characteristics**	
Cytology, n (%)	
- ASCUS	192 (36.6)
- LSIL	332 (63.4)
HPV status (multiple choice, n = 321) ^a^, n (%)	
- High-risk (HR)	212 (40.5)
- Possible HR	15 (2.9)
- Low-risk (LR)	105 (20.0)
- Negative	31 (5.9)
- HPV16 and/or HPV18 ^b^	57 (HR-HPV n = 212, 26.9)
**Vaccination and Lifestyle**	
HPV vaccinated, n (%)	121 (23.1)
Unvaccinated, n (%)	403 (76.9)
**Smoking status, n (%)**	
- Current smoker	170 (32.4)
- Ex-smoker	76 (14.5)
- Non-smoker	278 (53.1)
**Sexual Behavior**	
Partners in last month (n = 434), mean (SD)	1.0 (0.7)
Sexual contacts per month (n = 271), mean (SD)	6.9 (5.2)
Use of condoms within the last month, n = 524 (%)	Always 48 (9.2)
	Sometimes 154 (29.4)
	Never 106 (20.2)
	Not applicable 216 (41.2)
**Biopsy Results (n = 137) ^c^**	
- Negative, n (%)	4 (0.8)
- Inflammatory, n (%)	8 (1.5)
- Indicates HPV, n (%)	18 (3.4)
- CIN1, n (%)	107 (20.4)
**Relevant disease and/or surgery (n = 48) ^d^**	
Gynecological	37 (7.1)
Respiratory	1 (0.2)
Gastric	1 (0.2)
Thyroids	5 (0.9)
Others (psoriatic arthritis, kidney transplant, Dubin Johnson Syndrome)	4 (0.8)
**Relevant gynecological disease/surgery, n = 37 (%)**	
External Genital Warts removal	2 (0.4)
LGSIL, ASCUS, cervical lesions, HPV-positive results	6 (1.1)
Endometrial/endocervix polyp	2 (0.4)
Diagnostic curettage for climacteric uterine bleeding	1 (0.2)
Cervical cryotherapy	1 (0.2)
Cervical conization	19 (3.6)
Cervical biopsy	5 (0.9)
Bilateral salpingectomy	1 (0.2)

ASCUS, atypical squamous cells of undetermined significance; CIN, cervical intraepithelial neoplasia; HPV, human papillomavirus; HR, high-risk; LSIL, low-grade squamous intraepithelial lesion; SD, standard deviation. ^a^ HPV testing data available for 321 patients, of which 212 patients reported HR-HPV-positive test (40.5% of total sample). Calculated from total sample (n = 524). ^b^ HPV16 and/or HPV18 were calculated based on total HR-HPV (n = 212). ^c^ Percentages for biopsy results calculated from total sample (n = 524); 387 patients (73.9%) did not have baseline biopsy. ^d^ Percentages for relevant disease/surgery calculated from total sample (n = 524); 476 patients (90.8%) did not have baseline previous relevant disease/surgical procedures.

**Table 2:** Descriptive statistics on level of satisfaction on Visit 2 (6 months) and Visit 3 (12 months).

Visit	n	Valid %	Mean	Median	SD	Min	Max	Range	IQR
6 months	491	100.0	8.3	9	1.7	2	10	8	2
12 months	115	100.0	8.6	9	1.6	3	10	7	2

SD, standard deviation.

## Data Availability

The data presented in this study are available on request from the corresponding author due to participant confidentiality, data protection requirements, and sponsor ownership of the data.

## Supplementary Materials

The following supporting information can be downloaded at https://www.mdpi.com/article/10.3390/cancers18172762/s1. Table S1. List of participating sites (45 hospitals and private clinics across Greece). Table S2. Local Ethics Committees names and approval numbers across Greece.
