# Supplementary material for: Clinical Outcomes of a Multi-Ingredient Coriolus versicolor-Based Vaginal Gel in Greek Women with Low-Grade Cervical Lesions: The PAPILOBS-GR Real-Life Prospective Study
Source: Cancers (Basel). 2026 Aug 25;18(17):2762. doi: 10.3390/cancers18172762 (PMC13565064; doi:10.3390/cancers18172762)
Supplement: Supplementary file 1 [file cancers-18-02762-s001.zip › cancers-4487007-supplementary.pdf]

Supplementary Materials

Supplementary Table S1. List of participating sites (45 hospitals and private clinics across Greece).

Site Names (Institution, Clinic)	Principal (PI) /Sub (SI)- Investigators Names (First, Surname)
**Public Hospitals Names**	
General Hospital "Elena Venizelou", Obstetrics and Gynecology Department, Athens, Greece	Stavros Kanakakis (PI)<br>Labros Dritsoulas (SI)<br>Dimitrios Mostrous (SI)
University General Hospital of Patras "Panagia H Voitheia", Obstetrics and Gynecology Clinic, Patra, Greece	Georgios Michail (PI)
General Hospital of Argolida-Argos Nursing Unit, Obstetrics and Gynecology Clinic, Argos, Greece	Konstantinos Chronopoulos (PI)<br>Ifigeneia Papadimitriou (SI)
General Hospital of Ioannina "G. Hatzikosta", Obstetrics and Gynecology Clinic, Ioannina, Greece	Christodoulos Akrivis (PI)<br>Zoi Anastasiadi (SI)
General Hospital of Ptolemaida "Bodosakio", Obstetrics and Gynecology Clinic, Prolemaida, Greece	Kalliopi Antoniadou (PI)
University General Hospital of Larissa, Obstetrics and Gynecology Clinic, Larisa, Greece	Alexandros Daponte (PI)<br>George Valasoulis (SI)
General Hospital of Thessaloniki "Hippokration", 2nd University Obstetrics and Gynecology Clinic, Thessaloniki, Greece	Konstantinos Dinas (PI)<br>Georgios Pratilas (SI)<br>Nikolaos Tsampazis (SI)<br>Anastasios Liberis (SI)
General Hospital of Agios Nikolaos Crete, Obstetrics and Gynecology Department, Crete, Greece	Marinos Nikolaou (PI)
University General Hospital of Ioannina, Obstetrics and Gynecology Clinic, Ioannina, Greece	Evangelos Paraskevaidis (PI)<br>Antonios Athanasiou (SI)<br>Thomas Tsiantis (SI)
**Private Hospital Name**	
IASO General Clinic (IASO Hospital), Department of Obstetrics and Gynecology, Athens, Greece	Evripidis Bilirakis (PI)
**Private sites**	
**Private Obstetrics and Gynecology Sites**	
Private site, Attica, Greece	Dimitra Pouliou (PI)
Private site, Attica, Greece	Nikolaos Skabardonis (PI)
Private site, Attica, Greece	Konstantinos Sfakianoudis (PI)
Private site, Attica, Greece	Alkiviadis Tyrogiannis (PI)
Private site, Attica, Greece	Dimitrios Ftoulis (PI)
Private site, Attica, Greece	Dimitra Chrysagi (PI)
Private site, Volos, Greece	Vasilios Giaslakiotis (PI)
Private site, Larissa, Greece	Antonios Garas (PI)
Private site, Thessaloniki, Greece	Emmanouil Divanidis (PI)
Private site, Chania, Greece	Angeliki Zolindaki (PI)
Private site, Arta, Greece	Konstantina Katsoula (PI)
Private site, Thessaloniki, Greece	Sara Mameletzi (PI)
Private site, Larissa, Greece	Maria Manou (PI)
Private site, Heraklion Crete, Greece	Mara Marazaki (PI)
Private site, Drama, Greece	Despina Michailidou (PI)
Private site, Preveza, Greece	Georgios Bakolas (PI)

Private site, Thessaloniki, Greece	Evangelos Babas (PI)
Private site, Kavala, Greece	Athanasios Barbagiannis (PI)
Private site, Larissa, Greece	Alexandros Dafopoulos (PI)
Private site, Thessaloniki, Greece	Kimon Chatzistamatiou (PI)
Private site, Kozani, Greece	Dimitra Chlioumpi (PI)
Private site, Athens, Greece	Kalliopi Pappa (PI)
Private site, Attica, Greece	Eleftherios Anastasakis (PI)
Private site, Attica, Greece	Ilias Armatas (PI)
Private site, Livadeia, Greece	Aikaterini Vagou (PI)
Private site, Attica, Greece	Theodoros Vassiliadis (PI)
Private site, Attica, Greece	Nikolaos Evangelinakis (PI)
Private site, Attica, Greece	Panagiotis Thomopoulos (PI)
Private site, Attica, Greece	Theofanis Katerinakis (PI)
Private site, Attica, Greece	Stylianos Kogeorgos (PI)
Private site, Lamia, Greece	Athanasios Kontopanos (PI)
Private site, Attica, Greece	Evangelia Maragoudaki (PI)
Private site, Attica, Greece	Ismini Mavrikou (PI)
Private site, Korinthos, Greece	Evgenia Xarchoulakou (PI)
Private site, Korinthos, Greece	Anna Refaraci Papaioannou (PI)

Supplementary Table S2. Local Ethics Committees names and approval numbers across Greece.

Local Ethics Committees names	Approval reg. numbers	Ethic approval date.
Local Ethics Committee of General Hospital "Elena Venizelou"	191/2021	28-Sep-2021
Local Ethics Committee of University General Hospital of Patras “Panagia H Voitheia”	24563/2021	09-Sep-2021
Local Ethics Committee of General Hospital of Argolida-Argos Nursing Unit	11579/2021	04-Aug-2021
Local Ethics Committee of General Hospital of Ioannina “G. Hatzikosta”	12/2021	24-Sep-2021
Local Ethics Committee of General Hospital of Ptolemaida “Bodosakio”	60/2021	20-Jul-2021
Local Ethics Committee of University General Hospital of Larissa	26801/2021	20-Jul-2021
Local Ethics Committee of General Hospital of Thessaloniki “Hippokration”	31965/2021	20-Oct-2021
Local Ethics Committee of General Hospital of Agios Nikolaos, Crete	68/2021	29-Jul-2021
Local Ethics Committee of University General Hospital of Ioannina	21/2021	05-Nov-2021
Local Ethics Committee of IASO General Clinic (Private Hospital)	10/11/2021	09-Nov-2021
